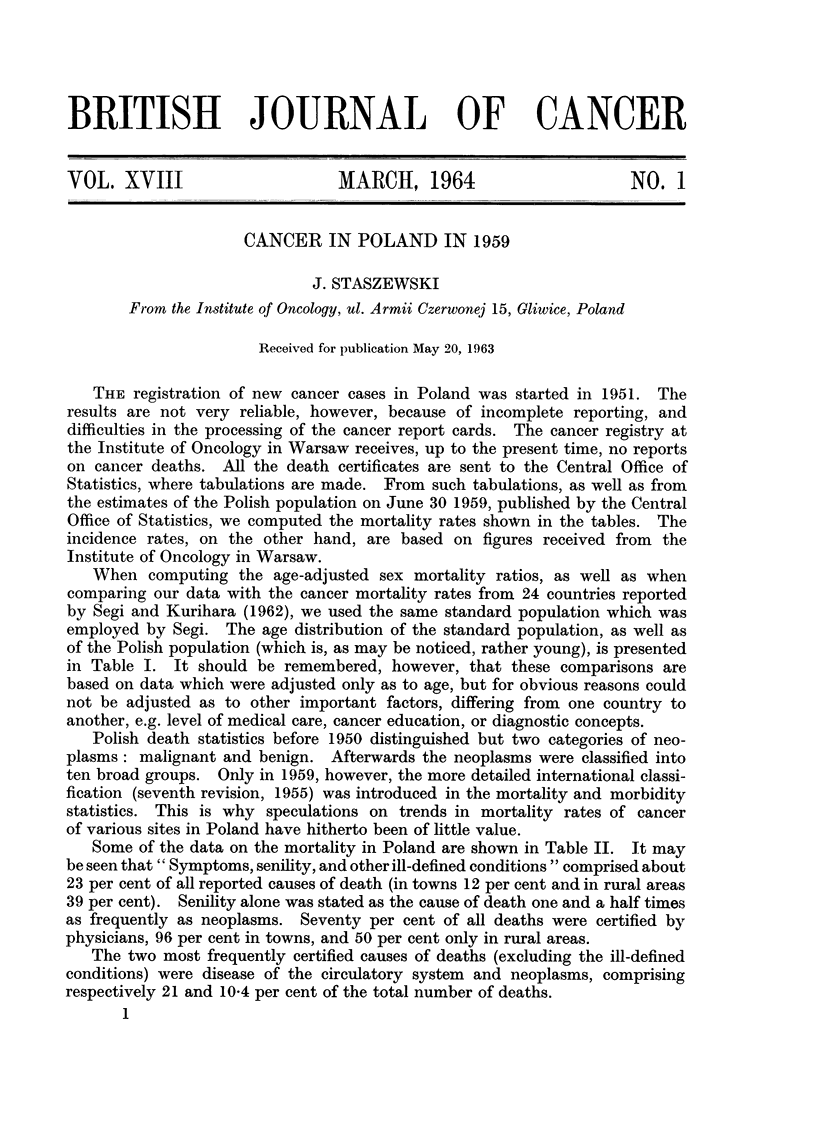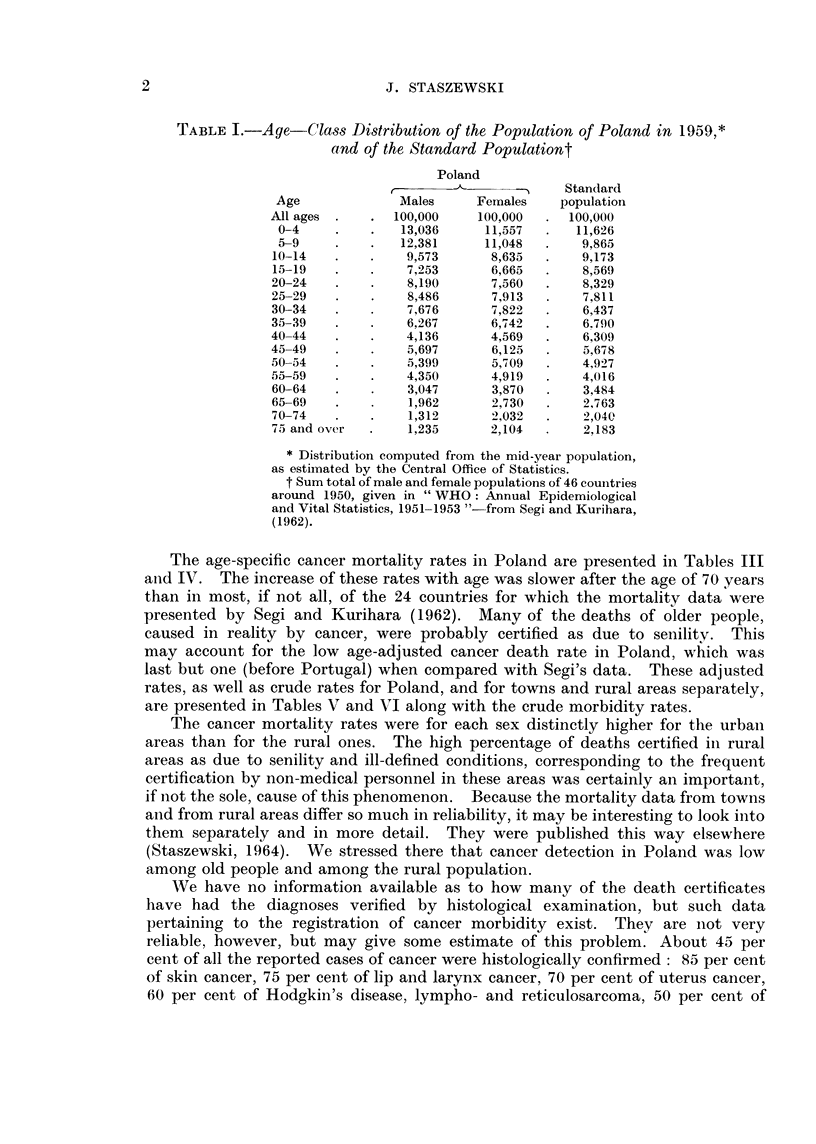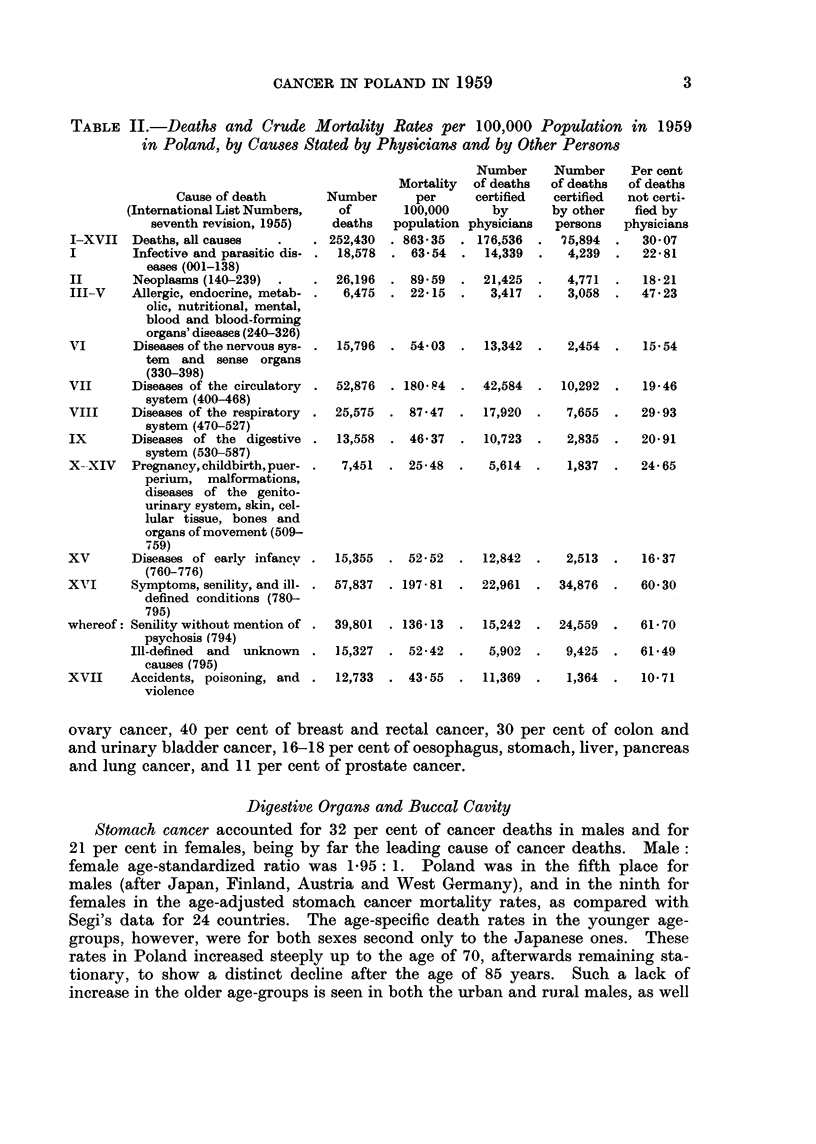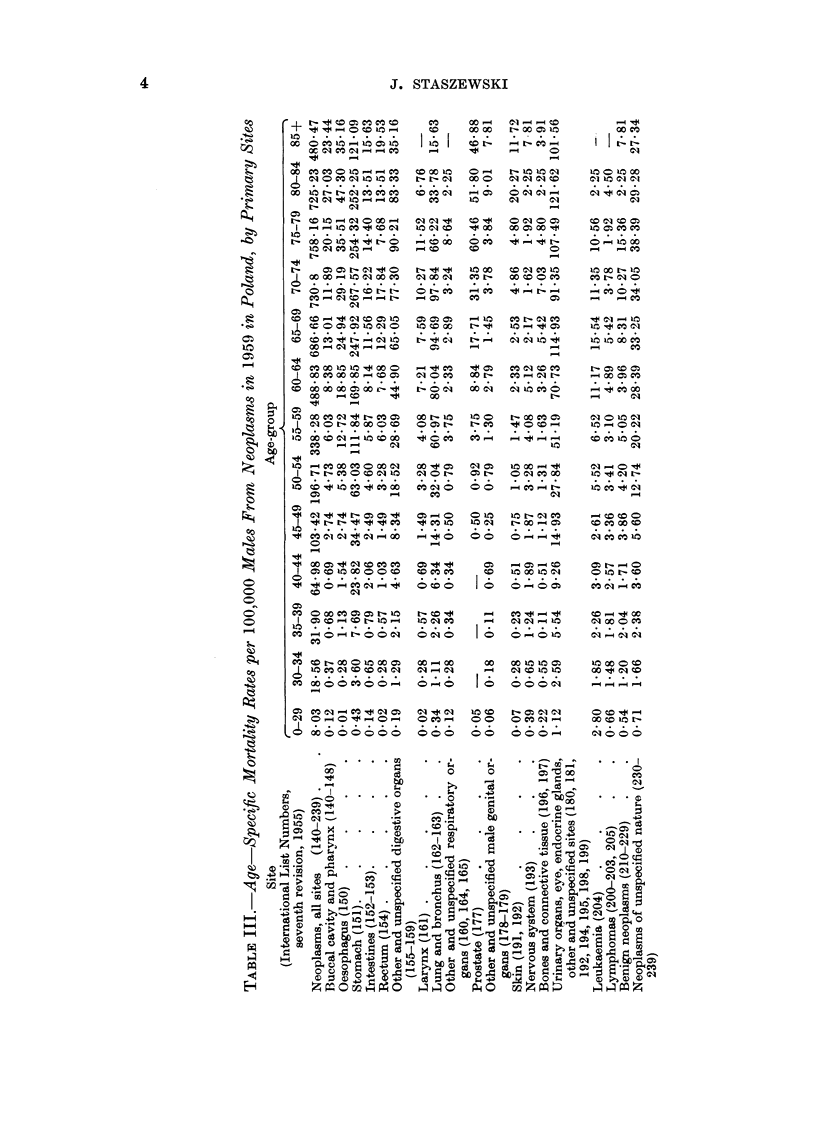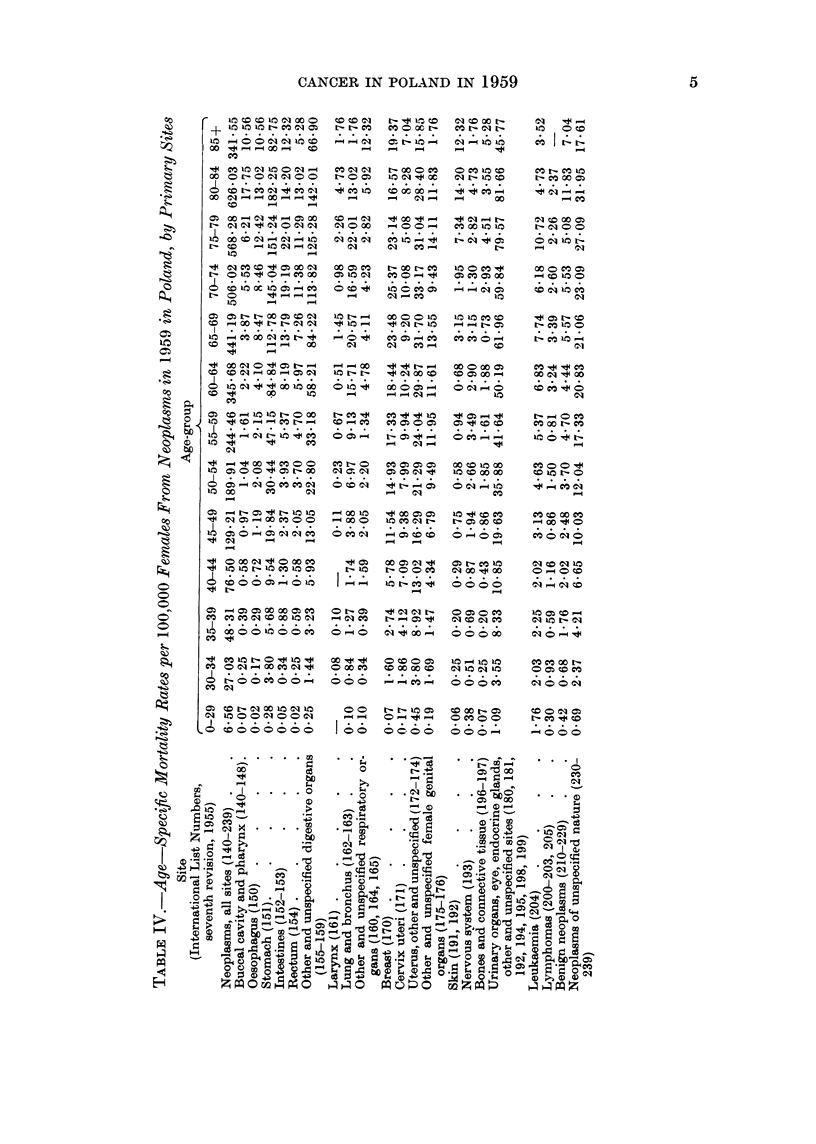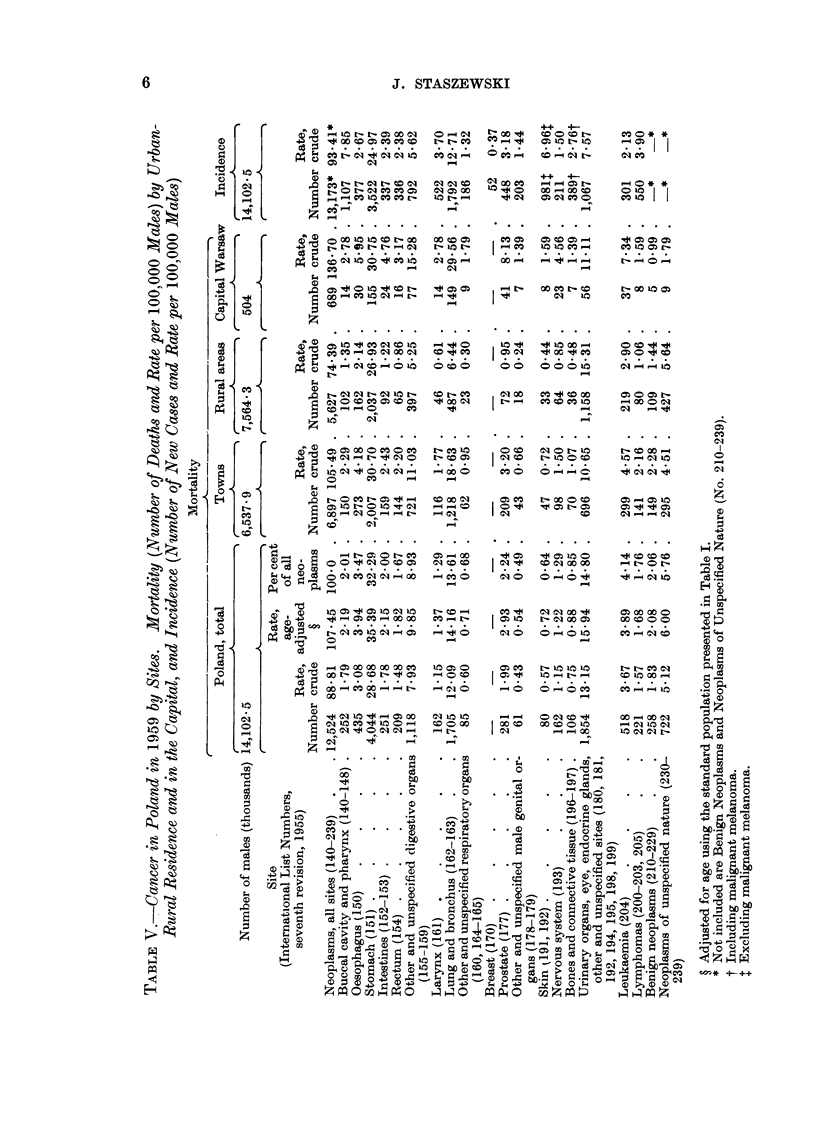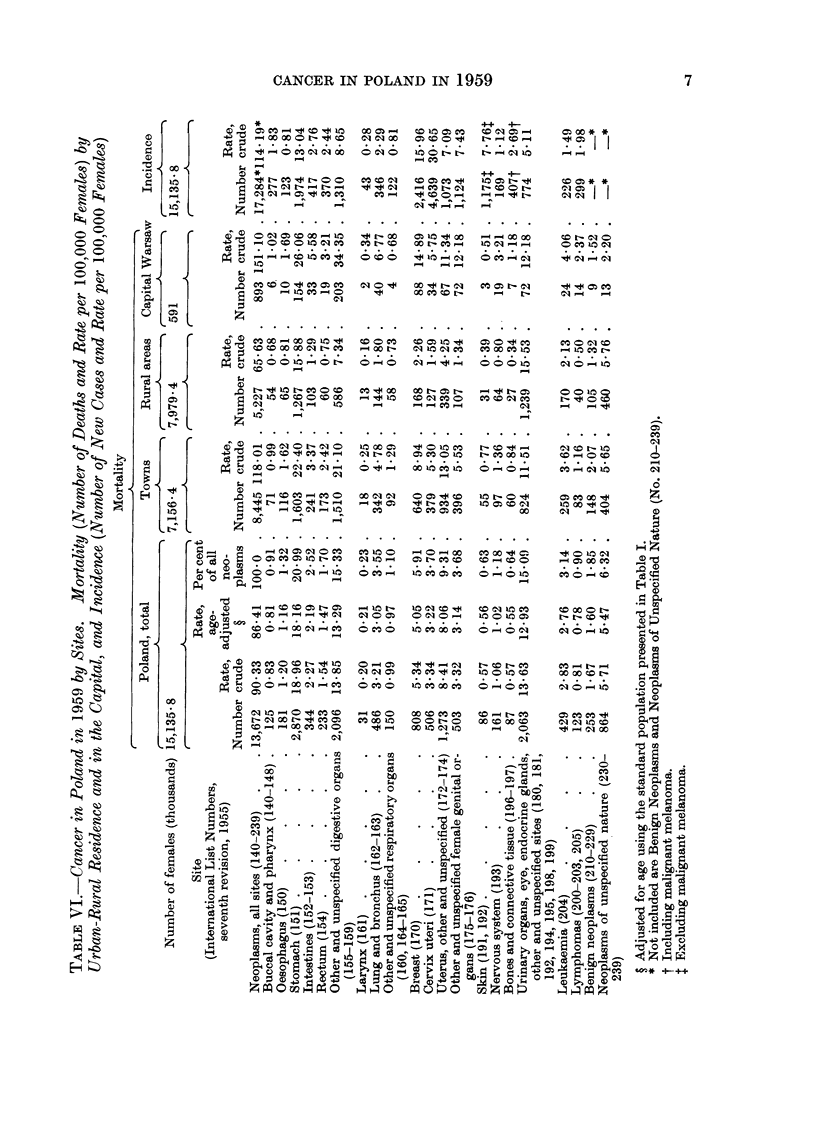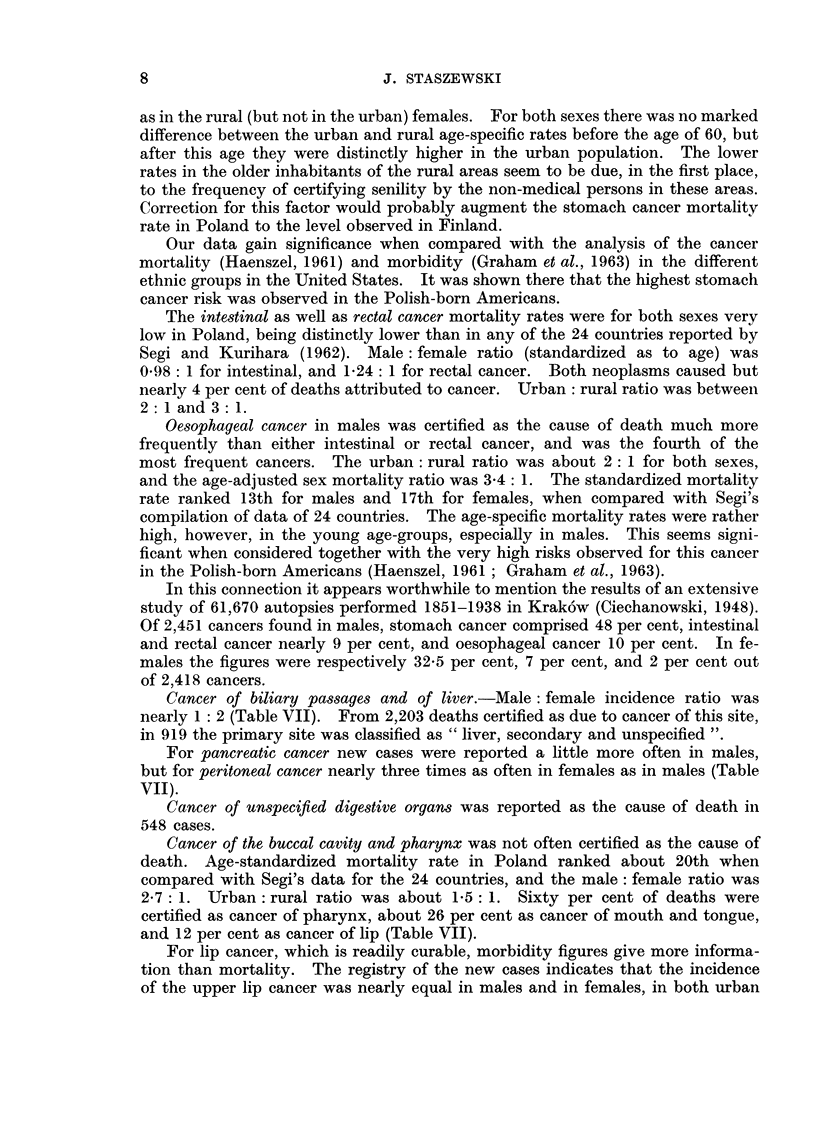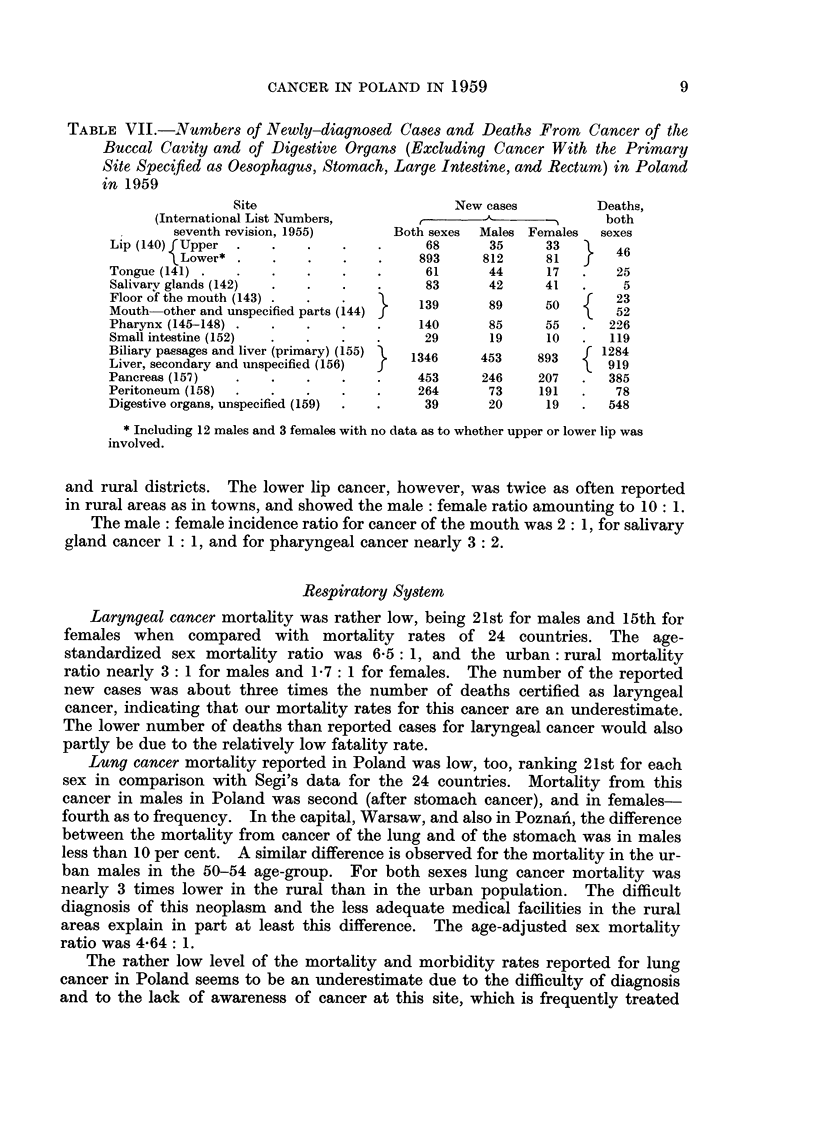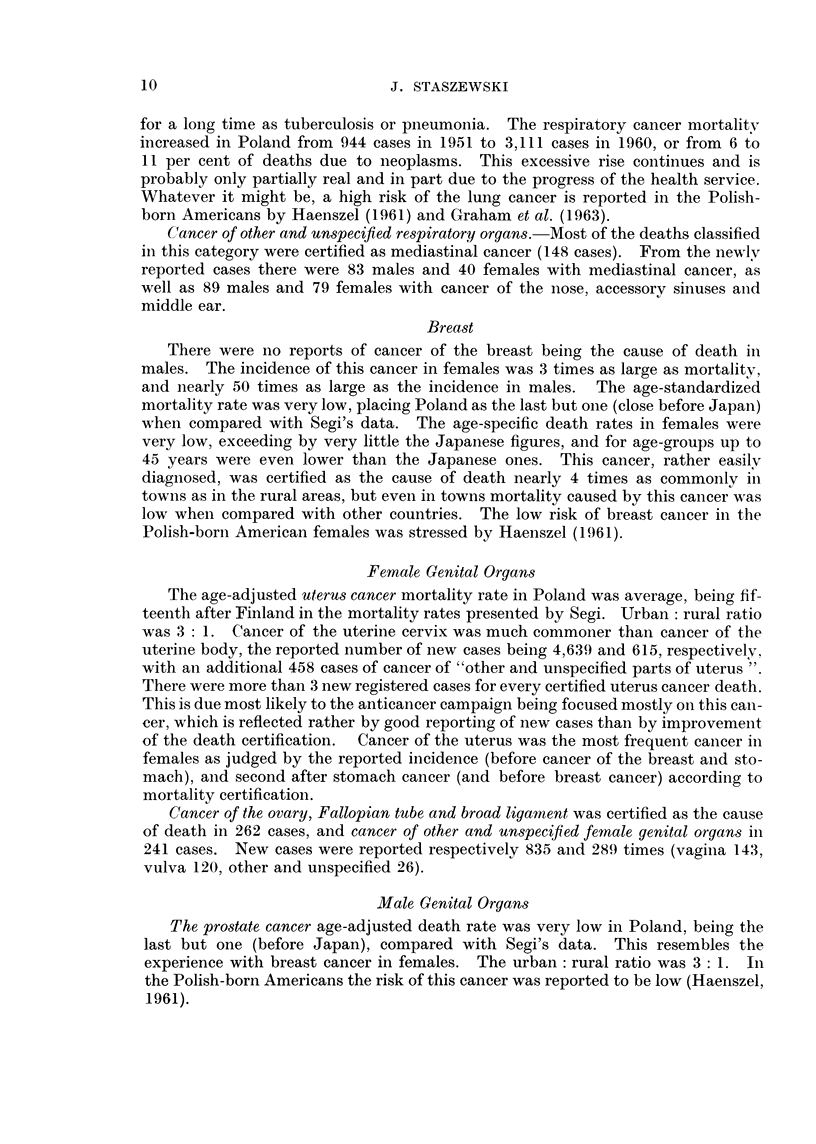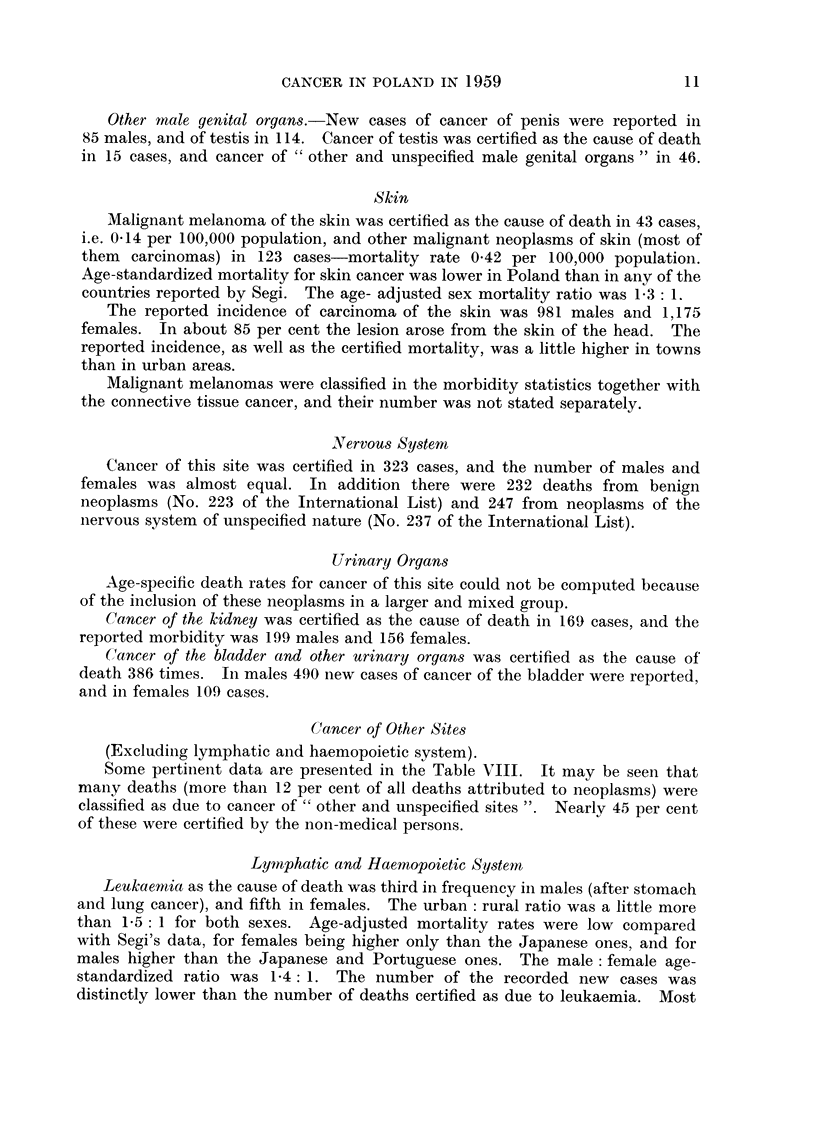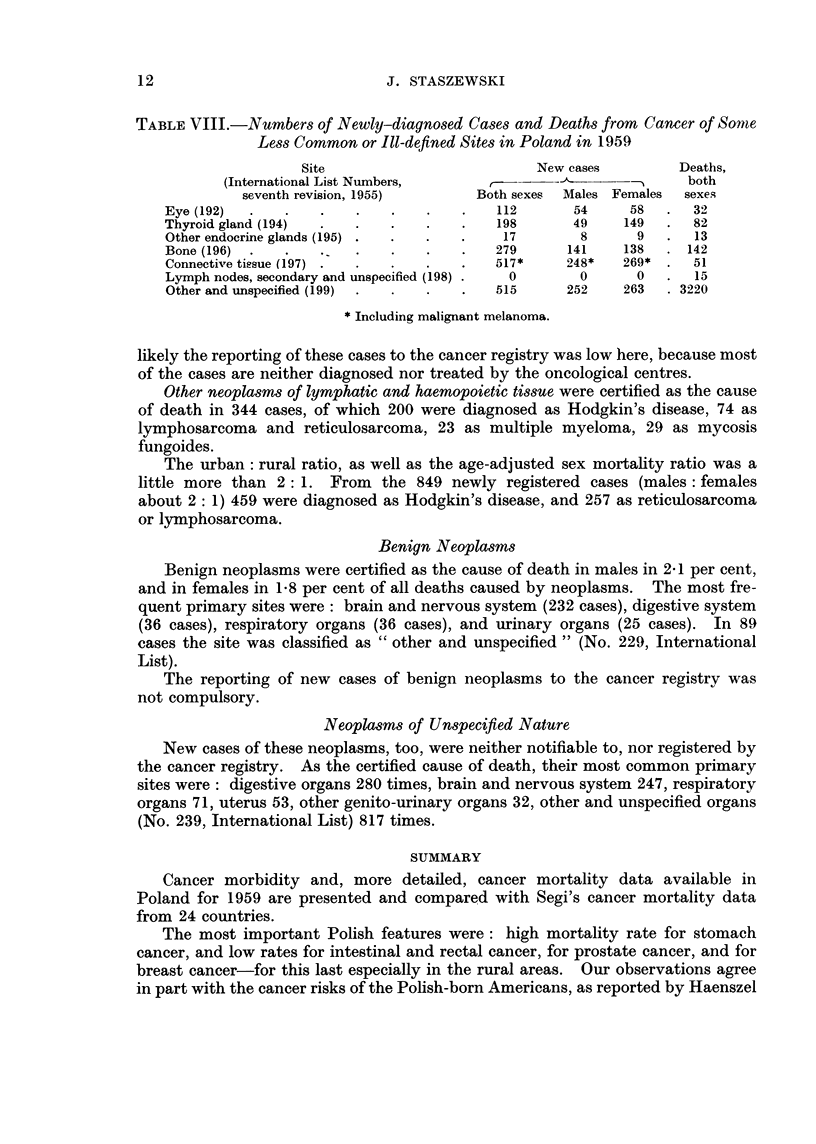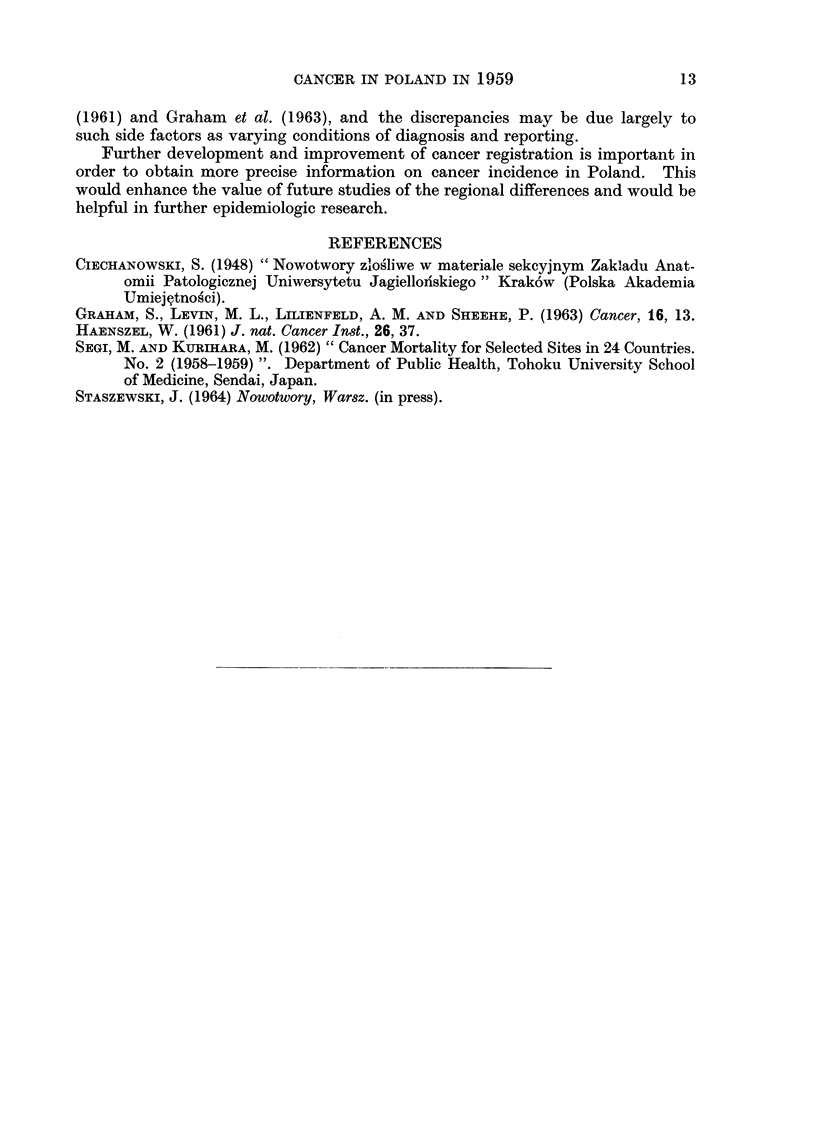# Cancer in Poland in 1959

**DOI:** 10.1038/bjc.1964.1

**Published:** 1964-03

**Authors:** J. Staszewski


### BRITISH JOURNAL OF CANCER

VOL. XVIII MARCH, 1964 NO. 1

### CANCER IN POLAND IN 1959

J. STASZEWSKI

*From the Institute of Oncology, ul. Armii Czerwonej* 15, *Gliwice, Poland*

Received for publication May 20, 1963

THE registration of new cancer cases in Poland was started in 1951. The results are not very reliable, however, because of incomplete reporting, and difficulties in the processing of the cancer report cards. The cancer registry at the Institute of Oncology in Warsaw receives, up to the present time, no reports on cancer deaths. All the death certificates are sent to the Central Office of Statistics, where tabulations are made. From such tabulations, as well as from the estimates of the Polish population on June 30 1959, published by the Central Office of Statistics, we computed the mortality rates shown in the tables. The incidence rates, on the other hand, are based on figures received from the Institute of Oncology in Warsaw.

When computing the age-adjusted sex mortality ratios, as well as when comparing our data with the cancer mortality rates from 24 countries reported by Segi and Kurihara (1962), we used the same standard population which was employed by Segi. The age distribution of the standard population, as well as of the Polish population (which is, as may be noticed, rather young), is presented in Table I. It should be remembered, however, that these comparisons are based on data which were adjusted only as to age, but for obvious reasons could not be adjusted as to other important factors, differing from one country to another, e.g. level of medical care, cancer education, or diagnostic concepts.

Polish death statistics before 1950 distinguished but two categories of neoplasms : malignant and benign. Afterwards the neoplasms were classified into ten broad groups. Only in 1959, however, the more detailed international classification (seventh revision, 1955) was introduced in the mortality and morbidity statistics. This is why speculations on trends in mortality rates of cancer of various sites in Poland have hitherto been of little value.

Some of the data on the mortality in Poland are shown in Table II. It may be seen that " Symptoms, senility, and other ill-defined conditions " comprised about 23 per cent of all reported causes of death (in towns 12 per cent and in rural areas 39 per cent). Senility alone was stated as the cause of death one and a half times as frequently as neoplasms. Seventy per cent of all deaths were certified by physicians, 96 per cent in towns, and 50 per cent only in rural areas.

The two most frequently certified causes of deaths (excluding the ill-defined conditions) were disease of the circulatory system and neoplasms, comprising respectively 21 and 10·4 per cent of the total number of deaths.

TABLE I.—*Age—Class Distribution of the Population of Poland in* 1959,* *and of the Standard Population*†

| Age | Poland: Males | Poland: Females | Standard population |
|---|---|---|---|
| All ages | 100,000 | 100,000 | 100,000 |
| 0–4 | 13,036 | 11,557 | 11,626 |
| 5–9 | 12,381 | 11,048 | 9,865 |
| 10–14 | 9,573 | 8,635 | 9,173 |
| 15–19 | 7,253 | 6,665 | 8,569 |
| 20–24 | 8,190 | 7,560 | 8,329 |
| 25–29 | 8,486 | 7,913 | 7,811 |
| 30–34 | 7,676 | 7,822 | 6,437 |
| 35–39 | 6,267 | 6,742 | 6.790 |
| 40–44 | 4,136 | 4,569 | 6.309 |
| 45–49 | 5,697 | 6,125 | 5,678 |
| 50–54 | 5,399 | 5,709 | 4,927 |
| 55–59 | 4,350 | 4,919 | 4,016 |
| 60–64 | 3,047 | 3,870 | 3,484 |
| 65–69 | 1,962 | 2,730 | 2,763 |
| 70–74 | 1,312 | 2,032 | 2,040 |
| 75 and over | 1,235 | 2,104 | 2,183 |

* Distribution computed from the mid-year population, as estimated by the Central Office of Statistics.

† Sum total of male and female populations of 46 countries around 1950, given in " WHO : Annual Epidemiological and Vital Statistics, 1951–1953 "—from Segi and Kurihara, (1962).

The age-specific cancer mortality rates in Poland are presented in Tables III and IV. The increase of these rates with age was slower after the age of 70 years than in most, if not all, of the 24 countries for which the mortality data were presented by Segi and Kurihara (1962). Many of the deaths of older people, caused in reality by cancer, were probably certified as due to senility. This may account for the low age-adjusted cancer death rate in Poland, which was last but one (before Portugal) when compared with Segi's data. These adjusted rates, as well as crude rates for Poland, and for towns and rural areas separately, are presented in Tables V and VI along with the crude morbidity rates.

The cancer mortality rates were for each sex distinctly higher for the urban areas than for the rural ones. The high percentage of deaths certified in rural areas as due to senility and ill-defined conditions, corresponding to the frequent certification by non-medical personnel in these areas was certainly an important, if not the sole, cause of this phenomenon. Because the mortality data from towns and from rural areas differ so much in reliability, it may be interesting to look into them separately and in more detail. They were published this way elsewhere (Staszewski, 1964). We stressed there that cancer detection in Poland was low among old people and among the rural population.

We have no information available as to how many of the death certificates have had the diagnoses verified by histological examination, but such data pertaining to the registration of cancer morbidity exist. They are not very reliable, however, but may give some estimate of this problem. About 45 per cent of all the reported cases of cancer were histologically confirmed : 85 per cent of skin cancer, 75 per cent of lip and larynx cancer, 70 per cent of uterus cancer, 60 per cent of Hodgkin's disease, lympho- and reticulosarcoma, 50 per cent of

TABLE II.—*Deaths and Crude Mortality Rates per* 100,000 *Population in* 1959 *in Poland, by Causes Stated by Physicians and by Other Persons*

| | Cause of death (International List Numbers, seventh revision, 1955) | Number of deaths | Mortality per 100,000 population | Number of deaths certified by physicians | Number of deaths certified by other persons | Per cent of deaths not certified by physicians |
|---|---|---|---|---|---|---|
| I–XVII | Deaths, all causes | 252,430 | 863·35 | 176,536 | 75,894 | 30·07 |
| I | Infective and parasitic diseases (001–138) | 18,578 | 63·54 | 14,339 | 4,239 | 22·81 |
| II | Neoplasms (140–239) | 26,196 | 89·59 | 21,425 | 4,771 | 18·21 |
| III–V | Allergic, endocrine, metabolic, nutritional, mental, blood and blood-forming organs' diseases (240–326) | 6,475 | 22·15 | 3,417 | 3,058 | 47·23 |
| VI | Diseases of the nervous system and sense organs (330–398) | 15,796 | 54·03 | 13,342 | 2,454 | 15·54 |
| VII | Diseases of the circulatory system (400–468) | 52,876 | 180·84 | 42,584 | 10,292 | 19·46 |
| VIII | Diseases of the respiratory system (470–527) | 25,575 | 87·47 | 17,920 | 7,655 | 29·93 |
| IX | Diseases of the digestive system (530–587) | 13,558 | 46·37 | 10,723 | 2,835 | 20·91 |
| X–XIV | Pregnancy, childbirth, puerperium, malformations, diseases of the genito-urinary system, skin, cellular tissue, bones and organs of movement (509–759) | 7,451 | 25·48 | 5,614 | 1,837 | 24·65 |
| XV | Diseases of early infancy (760–776) | 15,355 | 52·52 | 12,842 | 2,513 | 16·37 |
| XVI | Symptoms, senility, and ill-defined conditions (780–795) | 57,837 | 197·81 | 22,961 | 34,876 | 60·30 |
| whereof: | Senility without mention of psychosis (794) | 39,801 | 136·13 | 15,242 | 24,559 | 61·70 |
| | Ill-defined and unknown causes (795) | 15,327 | 52·42 | 5,902 | 9,425 | 61·49 |
| XVII | Accidents, poisoning, and violence | 12,733 | 43·55 | 11,369 | 1,364 | 10·71 |

ovary cancer, 40 per cent of breast and rectal cancer, 30 per cent of colon and and urinary bladder cancer, 16–18 per cent of oesophagus, stomach, liver, pancreas and lung cancer, and 11 per cent of prostate cancer.

### *Digestive Organs and Buccal Cavity*

*Stomach cancer* accounted for 32 per cent of cancer deaths in males and for 21 per cent in females, being by far the leading cause of cancer deaths. Male : female age-standardized ratio was 1·95 : 1. Poland was in the fifth place for males (after Japan, Finland, Austria and West Germany), and in the ninth for females in the age-adjusted stomach cancer mortality rates, as compared with Segi's data for 24 countries. The age-specific death rates in the younger age-groups, however, were for both sexes second only to the Japanese ones. These rates in Poland increased steeply up to the age of 70, afterwards remaining stationary, to show a distinct decline after the age of 85 years. Such a lack of increase in the older age-groups is seen in both the urban and rural males, as well

† Including malignant melanoma.

‡ Excluding malignant melanoma.

† Including malignant melanoma.

‡ Excluding malignant melanoma.

as in the rural (but not in the urban) females. For both sexes there was no marked difference between the urban and rural age-specific rates before the age of 60, but after this age they were distinctly higher in the urban population. The lower rates in the older inhabitants of the rural areas seem to be due, in the first place, to the frequency of certifying senility by the non-medical persons in these areas. Correction for this factor would probably augment the stomach cancer mortality rate in Poland to the level observed in Finland.

Our data gain significance when compared with the analysis of the cancer mortality (Haenszel, 1961) and morbidity (Graham *et al.*, 1963) in the different ethnic groups in the United States. It was shown there that the highest stomach cancer risk was observed in the Polish-born Americans.

The *intestinal* as well as *rectal cancer* mortality rates were for both sexes very low in Poland, being distinctly lower than in any of the 24 countries reported by Segi and Kurihara (1962). Male : female ratio (standardized as to age) was 0·98 : 1 for intestinal, and 1·24 : 1 for rectal cancer. Both neoplasms caused but nearly 4 per cent of deaths attributed to cancer. Urban : rural ratio was between 2 : 1 and 3 : 1.

*Oesophageal cancer* in males was certified as the cause of death much more frequently than either intestinal or rectal cancer, and was the fourth of the most frequent cancers. The urban : rural ratio was about 2 : 1 for both sexes, and the age-adjusted sex mortality ratio was 3·4 : 1. The standardized mortality rate ranked 13th for males and 17th for females, when compared with Segi's compilation of data of 24 countries. The age-specific mortality rates were rather high, however, in the young age-groups, especially in males. This seems significant when considered together with the very high risks observed for this cancer in the Polish-born Americans (Haenszel, 1961 ; Graham *et al.*, 1963).

In this connection it appears worthwhile to mention the results of an extensive study of 61,670 autopsies performed 1851–1938 in Kraków (Ciechanowski, 1948). Of 2,451 cancers found in males, stomach cancer comprised 48 per cent, intestinal and rectal cancer nearly 9 per cent, and oesophageal cancer 10 per cent. In females the figures were respectively 32·5 per cent, 7 per cent, and 2 per cent out of 2,418 cancers.

*Cancer of biliary passages and of liver.*—Male : female incidence ratio was nearly 1 : 2 (Table VII). From 2,203 deaths certified as due to cancer of this site, in 919 the primary site was classified as " liver, secondary and unspecified ".

For *pancreatic cancer* new cases were reported a little more often in males, but for *peritoneal cancer* nearly three times as often in females as in males (Table VII).

*Cancer of unspecified digestive organs* was reported as the cause of death in 548 cases.

*Cancer of the buccal cavity and pharynx* was not often certified as the cause of death. Age-standardized mortality rate in Poland ranked about 20th when compared with Segi's data for the 24 countries, and the male : female ratio was 2·7 : 1. Urban : rural ratio was about 1·5 : 1. Sixty per cent of deaths were certified as cancer of pharynx, about 26 per cent as cancer of mouth and tongue, and 12 per cent as cancer of lip (Table VII).

For lip cancer, which is readily curable, morbidity figures give more information than mortality. The registry of the new cases indicates that the incidence of the upper lip cancer was nearly equal in males and in females, in both urban

TABLE VII.—*Numbers of Newly-diagnosed Cases and Deaths From Cancer of the Buccal Cavity and of Digestive Organs (Excluding Cancer With the Primary Site Specified as Oesophagus, Stomach, Large Intestine, and Rectum) in Poland in* 1959

| Site (International List Numbers, seventh revision, 1955) | New cases: Both sexes | Males | Females | Deaths, both sexes |
|---|---|---|---|---|
| Lip (140) Upper | 68 | 35 | 33 | 46 |
| Lip (140) Lower* | 893 | 812 | 81 | |
| Tongue (141) | 61 | 44 | 17 | 25 |
| Salivary glands (142) | 83 | 42 | 41 | 5 |
| Floor of the mouth (143) | 139 | 89 | 50 | 23 |
| Mouth—other and unspecified parts (144) | | | | 52 |
| Pharynx (145–148) | 140 | 85 | 55 | 226 |
| Small intestine (152) | 29 | 19 | 10 | 119 |
| Biliary passages and liver (primary) (155) | 1346 | 453 | 893 | 1284 |
| Liver, secondary and unspecified (156) | | | | 919 |
| Pancreas (157) | 453 | 246 | 207 | 385 |
| Peritoneum (158) | 264 | 73 | 191 | 78 |
| Digestive organs, unspecified (159) | 39 | 20 | 19 | 548 |

* Including 12 males and 3 females with no data as to whether upper or lower lip was involved.

and rural districts. The lower lip cancer, however, was twice as often reported in rural areas as in towns, and showed the male : female ratio amounting to 10 : 1.

The male : female incidence ratio for cancer of the mouth was 2 : 1, for salivary gland cancer 1 : 1, and for pharyngeal cancer nearly 3 : 2.

### *Respiratory System*

*Laryngeal cancer* mortality was rather low, being 21st for males and 15th for females when compared with mortality rates of 24 countries. The age-standardized sex mortality ratio was 6·5 : 1, and the urban : rural mortality ratio nearly 3 : 1 for males and 1·7 : 1 for females. The number of the reported new cases was about three times the number of deaths certified as laryngeal cancer, indicating that our mortality rates for this cancer are an underestimate. The lower number of deaths than reported cases for laryngeal cancer would also partly be due to the relatively low fatality rate.

*Lung cancer* mortality reported in Poland was low, too, ranking 21st for each sex in comparison with Segi's data for the 24 countries. Mortality from this cancer in males in Poland was second (after stomach cancer), and in females—fourth as to frequency. In the capital, Warsaw, and also in Poznań, the difference between the mortality from cancer of the lung and of the stomach was in males less than 10 per cent. A similar difference is observed for the mortality in the urban males in the 50–54 age-group. For both sexes lung cancer mortality was nearly 3 times lower in the rural than in the urban population. The difficult diagnosis of this neoplasm and the less adequate medical facilities in the rural areas explain in part at least this difference. The age-adjusted sex mortality ratio was 4·64 : 1.

The rather low level of the mortality and morbidity rates reported for lung cancer in Poland seems to be an underestimate due to the difficulty of diagnosis and to the lack of awareness of cancer at this site, which is frequently treated for a long time as tuberculosis or pneumonia. The respiratory cancer mortality increased in Poland from 944 cases in 1951 to 3,111 cases in 1960, or from 6 to 11 per cent of deaths due to neoplasms. This excessive rise continues and is probably only partially real and in part due to the progress of the health service. Whatever it might be, a high risk of the lung cancer is reported in the Polish-born Americans by Haenszel (1961) and Graham *et al.* (1963).

*Cancer of other and unspecified respiratory organs.*—Most of the deaths classified in this category were certified as mediastinal cancer (148 cases). From the newly reported cases there were 83 males and 40 females with mediastinal cancer, as well as 89 males and 79 females with cancer of the nose, accessory sinuses and middle ear.

### *Breast*

There were no reports of cancer of the breast being the cause of death in males. The incidence of this cancer in females was 3 times as large as mortality, and nearly 50 times as large as the incidence in males. The age-standardized mortality rate was very low, placing Poland as the last but one (close before Japan) when compared with Segi's data. The age-specific death rates in females were very low, exceeding by very little the Japanese figures, and for age-groups up to 45 years were even lower than the Japanese ones. This cancer, rather easily diagnosed, was certified as the cause of death nearly 4 times as commonly in towns as in the rural areas, but even in towns mortality caused by this cancer was low when compared with other countries. The low risk of breast cancer in the Polish-born American females was stressed by Haenszel (1961).

### *Female Genital Organs*

The age-adjusted *uterus cancer* mortality rate in Poland was average, being fifteenth after Finland in the mortality rates presented by Segi. Urban : rural ratio was 3 : 1. Cancer of the uterine cervix was much commoner than cancer of the uterine body, the reported number of new cases being 4,639 and 615, respectively, with an additional 458 cases of cancer of "other and unspecified parts of uterus". There were more than 3 new registered cases for every certified uterus cancer death. This is due most likely to the anticancer campaign being focused mostly on this cancer, which is reflected rather by good reporting of new cases than by improvement of the death certification. Cancer of the uterus was the most frequent cancer in females as judged by the reported incidence (before cancer of the breast and stomach), and second after stomach cancer (and before breast cancer) according to mortality certification.

*Cancer of the ovary, Fallopian tube and broad ligament* was certified as the cause of death in 262 cases, and *cancer of other and unspecified female genital organs* in 241 cases. New cases were reported respectively 835 and 289 times (vagina 143, vulva 120, other and unspecified 26).

### *Male Genital Organs*

*The prostate cancer* age-adjusted death rate was very low in Poland, being the last but one (before Japan), compared with Segi's data. This resembles the experience with breast cancer in females. The urban : rural ratio was 3 : 1. In the Polish-born Americans the risk of this cancer was reported to be low (Haenszel, 1961).

*Other male genital organs.*—New cases of cancer of penis were reported in 85 males, and of testis in 114. Cancer of testis was certified as the cause of death in 15 cases, and cancer of " other and unspecified male genital organs " in 46.

### *Skin*

Malignant melanoma of the skin was certified as the cause of death in 43 cases, i.e. 0·14 per 100,000 population, and other malignant neoplasms of skin (most of them carcinomas) in 123 cases—mortality rate 0·42 per 100,000 population. Age-standardized mortality for skin cancer was lower in Poland than in any of the countries reported by Segi. The age- adjusted sex mortality ratio was 1·3 : 1.

The reported incidence of carcinoma of the skin was 981 males and 1,175 females. In about 85 per cent the lesion arose from the skin of the head. The reported incidence, as well as the certified mortality, was a little higher in towns than in urban areas.

Malignant melanomas were classified in the morbidity statistics together with the connective tissue cancer, and their number was not stated separately.

### *Nervous System*

Cancer of this site was certified in 323 cases, and the number of males and females was almost equal. In addition there were 232 deaths from benign neoplasms (No. 223 of the International List) and 247 from neoplasms of the nervous system of unspecified nature (No. 237 of the International List).

### *Urinary Organs*

Age-specific death rates for cancer of this site could not be computed because of the inclusion of these neoplasms in a larger and mixed group.

*Cancer of the kidney* was certified as the cause of death in 169 cases, and the reported morbidity was 199 males and 156 females.

*Cancer of the bladder and other urinary organs* was certified as the cause of death 386 times. In males 490 new cases of cancer of the bladder were reported, and in females 109 cases.

### *Cancer of Other Sites*

(Excluding lymphatic and haemopoietic system).

Some pertinent data are presented in the Table VIII. It may be seen that many deaths (more than 12 per cent of all deaths attributed to neoplasms) were classified as due to cancer of " other and unspecified sites ". Nearly 45 per cent of these were certified by the non-medical persons.

### *Lymphatic and Haemopoietic System*

*Leukaemia* as the cause of death was third in frequency in males (after stomach and lung cancer), and fifth in females. The urban : rural ratio was a little more than 1·5 : 1 for both sexes. Age-adjusted mortality rates were low compared with Segi's data, for females being higher only than the Japanese ones, and for males higher than the Japanese and Portuguese ones. The male : female age-standardized ratio was 1·4 : 1. The number of the recorded new cases was distinctly lower than the number of deaths certified as due to leukaemia. Most

TABLE VIII.—*Numbers of Newly-diagnosed Cases and Deaths from Cancer of Some Less Common or Ill-defined Sites in Poland in* 1959

| Site (International List Numbers, seventh revision, 1955) | New cases | | | Deaths, both sexes |
|---|---|---|---|---|
| | Both sexes | Males | Females | |
| Eye (192) | 112 | 54 | 58 | 32 |
| Thyroid gland (194) | 198 | 49 | 149 | 82 |
| Other endocrine glands (195) | 17 | 8 | 9 | 13 |
| Bone (196) | 279 | 141 | 138 | 142 |
| Connective tissue (197) | 517* | 248* | 269* | 51 |
| Lymph nodes, secondary and unspecified (198) | 0 | 0 | 0 | 15 |
| Other and unspecified (199) | 515 | 252 | 263 | 3220 |

likely the reporting of these cases to the cancer registry was low here, because most of the cases are neither diagnosed nor treated by the oncological centres.

*Other neoplasms of lymphatic and haemopoietic tissue* were certified as the cause of death in 344 cases, of which 200 were diagnosed as Hodgkin's disease, 74 as lymphosarcoma and reticulosarcoma, 23 as multiple myeloma, 29 as mycosis fungoides.

The urban : rural ratio, as well as the age-adjusted sex mortality ratio was a little more than 2 : 1. From the 849 newly registered cases (males : females about 2 : 1) 459 were diagnosed as Hodgkin's disease, and 257 as reticulosarcoma or lymphosarcoma.

### *Benign Neoplasms*

Benign neoplasms were certified as the cause of death in males in 2·1 per cent, and in females in 1·8 per cent of all deaths caused by neoplasms. The most frequent primary sites were : brain and nervous system (232 cases), digestive system (36 cases), respiratory organs (36 cases), and urinary organs (25 cases). In 89 cases the site was classified as " other and unspecified " (No. 229, International List).

The reporting of new cases of benign neoplasms to the cancer registry was not compulsory.

### *Neoplasms of Unspecified Nature*

New cases of these neoplasms, too, were neither notifiable to, nor registered by the cancer registry. As the certified cause of death, their most common primary sites were : digestive organs 280 times, brain and nervous system 247, respiratory organs 71, uterus 53, other genito-urinary organs 32, other and unspecified organs (No. 239, International List) 817 times.

### SUMMARY

Cancer morbidity and, more detailed, cancer mortality data available in Poland for 1959 are presented and compared with Segi's cancer mortality data from 24 countries.

The most important Polish features were : high mortality rate for stomach cancer, and low rates for intestinal and rectal cancer, for prostate cancer, and for breast cancer—for this last especially in the rural areas. Our observations agree in part with the cancer risks of the Polish-born Americans, as reported by Haenszel

(1961) and Graham *et al.* (1963), and the discrepancies may be due largely to such side factors as varying conditions of diagnosis and reporting.

Further development and improvement of cancer registration is important in order to obtain more precise information on cancer incidence in Poland. This would enhance the value of future studies of the regional differences and would be helpful in further epidemiologic research.

### REFERENCES

CIECHANOWSKI, S. (1948) " Nowotwory złośliwe w materiale sekcyjnym Zakładu Anatomii Patologicznej Uniwersytetu Jagiellońskiego " Kraków (Polska Akademia Umiejętności).

GRAHAM, S., LEVIN, M. L., LILIENFELD, A. M. AND SHEEHE, P. (1963) *Cancer*, **16**, 13.

HAENSZEL, W. (1961) *J. nat. Cancer Inst.*, **26**, 37.

SEGI, M. AND KURIHARA, M. (1962) " Cancer Mortality for Selected Sites in 24 Countries. No. 2 (1958–1959) ". Department of Public Health, Tohoku University School of Medicine, Sendai, Japan.

STASZEWSKI, J. (1964) *Nowotwory, Warsz.* (in press).